# A236 ASSOCIATION OF STOOL METABOLOMIC PROFILE AND MICROBIOME COMPOSITION RISK SCORE WITH FUTURE ONSET OF CROHN’S DISEASE

**DOI:** 10.1093/jcag/gwab049.235

**Published:** 2022-02-21

**Authors:** S Lee, J Raygoza Garay, W Turpin, M I Smith, A Goethel, A Griffiths, P Moayyedi, O Espin-Garcia, G Aumais, C N Bernstein, I Avni-Biron, M Cino, C Deslandres, I Dotan, W El-Matary, B G Feagan, D S Guttmen, H Q Huynh, J Hyams, K Jacobson, D R Mack, J Marshall, A Otley, R Panaccione, M S Silverberg, H Steinhart, D Turner, W Xu, K Croitoru

**Affiliations:** 1 Department of Gastroenterology, Mount Sinai Hospital, Toronto, ON, Canada; 2 University of Toronto Dalla Lana School of Public Health, Toronto, ON, Canada; 3 Hospital for Sick Children, Toronto, ON, Canada; 4 McMaster University, Hamilton, ON, Canada; 5 Uiversity of Manitoba, Winnipeg, MB, Canada; 6 Toronto Western Hospital, Toronto, ON, Canada; 7 Service de gastro-entérologie, CHU Sainte-Justine, Montréal, QC, Canada; 8 Western University Schulich School of Medicine & Dentistry, London, ON, Canada; 9 Pediatrics, University of alberta, Edmonton, AB, Canada; 10 BC Children’s Hospital, Vancouver, BC, Canada; 11 McMaster University Medical Centre, Hamilton, ON, Canada; 12 Pediatrics, Dalhousie University, Halifax, NS, Canada; 13 University of Calgary, Calgary, AB, Canada; 14 Hopital Maisonneuve-Rosemont, Montreal, QC, Canada; 15 Rabin Medical Center, Petah Tikva, Israel; 16 Connecticut Children’s Medical Center, Hartford, CT; 17 Children’s Hospital of Eastern Ontario, Ottawa, ON, Canada; 18 Shaare Zedek Medical Center, Jerusalem, Jerusalem, Israel; 19 University of Toronto, Toronto, ON, Canada

## Abstract

**Background:**

Microbial composition-based risk score (MRS) was recently developed and validated to predict future risk of developing Crohn’s disease (CD) among healthy first-degree relatives (FDR) of CD patients. We hypothesized that stool metabolomic profiles, some of which are linked to the gut microbiome, are associated with future risk of CD.

**Aims:**

To assess the association of stool metabolomic profile with onset of CD and to determine the correlation between stool metabolites and the MRS

**Methods:**

Healthy FDR of CD patients were recruited as part of the nested case-control cohort of the CCC-GEM Project. Healthy FDRs who later developed CD (n=56) were matched approximately 1:1 by age, sex, follow-up duration, and geographical location with control FDRs remaining healthy (n=66). Stool metabolomics were assessed using the Metabolon’s DiscoveryHD4™ platform, and the stool microbiome characterised by 16s rDNA amplicon sequencing. We fitted a multivariable conditional logistic regression model on the disease status as a function of individual stool metabolites. We additionally performed Spearman correlation between each stool metabolite and the MRS.

**Results:**

Among 1,029 stool metabolites that were analyzed, 79 were associated with future risk of CD (p<0.05); however, none remained significant after multiple testing correction (FDR correction). Considering the exploratory nature of this study with limited sample size, we focused on the top seven metabolites associated with CD onset (p<0.01). Of these, two stool metabolites (dimethylglycine, methylmyristate) were associated with increased risk of CD onset while five (cytosine, guanine, cytidine, hydroxyglutarate, nervonate) were associated with decreased risk of developing CD. The two metabolites positively associated with CD onset were positively correlated with the MRS, while the five metabolites negatively associated with CD onset, were negatively correlated with the MRS. Meanwhile, 24 stool metabolites had significant correlation with MRS (FDR-corrected p<0.2). Among those, a total of four stool metabolites (cytosine, guanine, methymyristate, cytidine) overlapped with the top seven stool metabolites associated with CD onset.

**Conclusions:**

Stool metabolite profiles may predict future risk of CD. A subset of these metabolites have significant correlation with the MRS with consistent direction of effect. This may suggest that stool metabolites mediate the putative effect of the gut microbiome on CD risk. Further validation in the full GEM cohort is warranted.

**Funding Agencies:**

CCC, CIHRThe Leona M. and Harry B. Helmsley Charitable Trust; Kenneth Croitoru is the recipient of the Canada Research Chair in Inflammatory Bowel Diseases; Sun-Ho Lee is a recipient of the Imagine/ CIHR/CAG Fellowship Award; Sun-Ho Lee, Juan Antonio Raygoza Garay, and Williams Turpin are recipients of fellowship awards from the Department of Medicine, Mount Sinai Hospital, Toronto, Canada.

